# A Lightweight Robot-View Visual Sensing Framework for CPU-Oriented License Plate Detection and Recognition in Mobile Robotic Scenarios

**DOI:** 10.3390/s26134170

**Published:** 2026-07-02

**Authors:** Ziyuan Wang, Juan Tang, Xinzheng Cao, Hui Shang

**Affiliations:** School of Mechanical and Automotive Engineering (School of Precision Manufacturing), Liaocheng University, Liaocheng 252000, China; m18365652689@163.com (Z.W.); 17663569306@163.com (X.C.); shanghui0307@gmail.com (H.S.)

**Keywords:** license plate detection and recognition, lightweight network, YOLOv8-MGL, SimAM-LPRNet, edge deployment, mobile inspection robots

## Abstract

Mobile inspection robots require reliable license plate detection and recognition under constrained computing resources, small-scale or distant imaging conditions, motion blur, and complex background interference. To address these coupled challenges, this paper proposes a lightweight robot-view visual sensing framework for CPU-oriented license plate perception. Instead of simply stacking network modules, the proposed framework follows a unified design principle of reducing redundant computation while compensating for task-critical visual information. In the detection stage, a YOLOv8-MGL detector is developed based on YOLOv8n by combining GhostC2f-based lightweight feature aggregation with LSKAlite-based contextual enhancement after the SPPF module. In the recognition stage, SimAM is embedded into LPRNet to enhance discriminative character responses under motion blur, low resolution, and local degradation without introducing additional learnable parameters. Experiments on the held-out EDRV-LP test set show that YOLOv8-MGL achieves 99.5% mAP_50_ and 71.1% mAP_50:95_, while reducing the number of parameters from 3.01 M to 2.77 M and GFLOPs from 8.1 to 7.5 compared with YOLOv8n. On a CPU-only Intel Xeon Platinum 8260C platform, YOLOv8-MGL achieves 23.98 FPS. SimAM-LPRNet improves the module-level cropped-plate recognition accuracy from 83.10% to 87.17%. To further examine system-level feasibility, a supplementary YOLOv8-MGL + CRNN-CTC pipeline is evaluated from raw images to final plate strings, achieving 91.0% exact recognition accuracy on the held-out EDRV-LP test set, 92.0% on a non-overlapping external CCPD subset, and 13.25 FPS for complete CPU-only processing. These results demonstrate that the proposed framework provides a favorable trade-off among model compactness, localization quality, recognition robustness, and CPU-oriented inference feasibility for mobile robotic inspection scenarios.

## 1. Introduction

With the continuous advancement of intelligent transportation systems and smart city development, mobile robotic visual perception has become an important task in intelligent transportation, mobile inspection, and public security scenarios [1,2]. Compared with fixed cameras, mobile robots and unmanned inspection vehicles provide stronger mobility, wider coverage, and greater deployment flexibility, making them suitable for campus management, road inspection, parking supervision, and autonomous security patrol [3]. In such systems, license plate perception is not merely an isolated recognition task, but a typical robot-view visual sensing problem that requires accurate object localization, robust character understanding, and efficient inference under constrained edge computing resources.

However, license plate perception in mobile robotic scenarios is considerably more challenging than recognition under fixed-view conditions. First, mobile robots usually operate on CPU-only or low-power embedded platforms, where available computational resources are limited and real-time inference is difficult to maintain [4]. Second, due to long-distance imaging and continuous robot motion, license plates may occupy relatively small regions in the captured frames, resulting in weak texture representation and feature degradation during network downsampling. Third, dynamic acquisition introduces motion blur, perspective distortion, illumination variation, partial occlusion, and complex background interference [5], which further affects both bounding-box localization and fine-grained character representation. Therefore, an effective robotic license plate sensing system should jointly consider lightweight inference, scale variation, and robustness against dynamic visual degradation.

In recent years, deep learning-based object detection and sequence recognition methods have been widely adopted in license plate perception systems. In particular, single-stage object detection algorithms represented by the YOLO family have achieved a favorable balance between detection accuracy and inference speed, and have become a mainstream technical route in intelligent transportation visual sensing tasks, including vehicle monitoring and license plate detection [6,7,8,9]. Although newer models such as YOLOv9 [10] and YOLOv10 [11] have shown strong performance on general-purpose object detection datasets, their structural complexity and deployment cost remain important concerns for mobile robotic edge platforms. For CPU-only or resource-constrained scenarios, directly pursuing larger or more complex detection networks may not necessarily lead to a better balance among accuracy, model complexity, and inference efficiency. Meanwhile, lightweight detectors may suffer from insufficient contextual modeling and degraded small-object representation under long-distance imaging conditions. Similarly, lightweight recognition networks such as LPRNet are suitable for edge deployment, but their native feature extractors lack explicit mechanisms for enhancing degraded character regions under motion blur, low resolution, and perspective distortion. These limitations indicate that license plate perception for mobile robots requires not only model lightweighting, but also coordinated feature enhancement tailored to scale-varying license plate regions and dynamically degraded character regions.

To address these issues, this paper proposes a lightweight robot-view visual sensing framework for CPU-oriented license plate detection and recognition in mobile robotic scenarios. The proposed framework is designed for mobile robotic inspection scenarios, where license plates are often captured from non-fixed viewpoints and are affected by long-distance imaging, motion blur, perspective distortion, illumination variation, and background interference. Different from conventional license plate recognition pipelines that mainly optimize detection or recognition accuracy alone, the proposed framework jointly considers lightweight detection, contextual enhancement for scale-varying license plate regions, degradation-aware character recognition, and CPU-oriented inference efficiency. Specifically, a YOLOv8-MGL detector is developed based on YOLOv8n. Ghost-based lightweight operations [4,12] are introduced into the feature aggregation neck to reduce redundant convolutional computation, while LSKAlite is inserted after the SPPF module to enhance the contextual representation of scale-varying and degraded license plate regions. In the recognition stage, SimAM is embedded into LPRNet to strengthen the feature representation of key character regions under motion blur, low resolution, and local degradation without introducing additional learnable parameters. To verify the effectiveness of the proposed method, a robot-view license plate dataset is constructed, and comprehensive evaluations of detection accuracy, recognition accuracy, model complexity, and CPU-only inference capability are conducted.

Accordingly, the proposed method is positioned as an edge-oriented robot-view visual sensing framework rather than a conventional license plate recognition pipeline. The design logic of the framework is consistent with the practical requirements of mobile inspection robots: reducing redundant computation for edge inference, enhancing contextual representation for scale-varying license plate regions, and improving degraded character representations caused by dynamic acquisition.

Based on this motivation, the main contributions of this paper are summarized as follows:

1. **A lightweight robot-view visual sensing framework for mobile robotic CPU-oriented deployment is proposed.** Unlike conventional license plate recognition pipelines that mainly focus on isolated detection or recognition accuracy, the proposed framework formulates license plate perception as a robot-view visual sensing problem under constrained computing resources. A decoupled detection-and-recognition architecture is designed to improve deployment flexibility and system-level inference feasibility in dynamic mobile inspection scenarios.
2. **An efficient YOLOv8-MGL detection network is developed for scale-varying license plate localization.** GhostC2f is introduced into the feature aggregation neck to reduce redundant convolutional computation, while LSKAlite is inserted after the SPPF module to enhance high-level contextual representation for scale-varying and degraded license plate regions. The resulting detection network improves the trade-off between model compactness and localization quality under stricter IoU thresholds.
3. **A SimAM-enhanced lightweight recognition network is designed to improve degraded character representation.** By integrating the parameter-free SimAM attention mechanism into LPRNet, the recognition network strengthens responses in critical character regions affected by motion blur, perspective distortion, and local low resolution without increasing learnable parameter overhead.
4. **A robot-view license plate dataset with held-out and supplementary system-level validation is provided.** The self-built EDRV-LP dataset is evaluated using a held-out test set rather than reporting final results only on the validation set. On this test set, YOLOv8-MGL achieves 99.5% mAP_50_ and 71.1% mAP_50:95_, with 2.77 M parameters, 7.5 GFLOPs, and 23.98 FPS on an Intel Xeon Platinum 8260C CPU-only platform. SimAM-LPRNet improves the module-level cropped-plate recognition accuracy from 83.10% to 87.17%. In addition, a supplementary YOLOv8-MGL + CRNN-CTC pipeline achieves 91.0% exact recognition accuracy on the held-out EDRV-LP test set and 92.0% on a non-overlapping external CCPD subset.

## 2. Related Work

### 2.1. Lightweight Object Detection for Edge Deployment

Object detection is a fundamental task in visual perception systems. Early two-stage detection methods, such as Fast R-CNN and Faster R-CNN [13], achieved high detection accuracy, but their relatively complex architectures and multi-stage inference procedures limit their applicability in real-time edge scenarios. In contrast, single-stage detectors represented by SSD [14] and YOLO [15] directly perform object localization and classification within a unified network, achieving a better balance between accuracy and inference speed. Therefore, they are more suitable for real-time visual perception tasks on resource-constrained platforms such as mobile robots and embedded edge devices [16].

Among existing single-stage detectors, YOLOv5, YOLOv7, and YOLOv8 have been widely adopted in engineering applications [17,18]. YOLOv8 is often selected as a baseline for lightweight improvement due to its concise network structure, anchor-free detection head, and favorable deployability. Although newer detectors such as YOLOv9 and YOLOv10 have shown competitive performance on general object detection tasks, their deployment cost and structural complexity still need to be considered when they are applied to mobile robotic edge platforms [10,11]. Therefore, how to reduce computational overhead while maintaining reliable detection accuracy remains an important issue for edge-oriented visual perception.

To improve inference efficiency, lightweight network designs have been extensively studied. MobileNet reduces computation through depthwise separable convolutions [19], while GhostNet generates additional feature maps through low-cost linear operations to reduce redundant convolutional computation [12]. Based on this idea, Ghost-YOLO introduces Ghost modules into the YOLO framework and combines feature enhancement strategies to improve the efficiency of edge-oriented object detection [4]. These studies demonstrate that lightweight feature extraction is effective for reducing model complexity. However, in mobile robot license plate detection, lightweight compression alone may weaken the representation of scale-varying and degraded license plate regions. Therefore, the detector in this paper combines Ghost-based redundancy reduction with contextual feature compensation to better balance detection accuracy and computational efficiency.

### 2.2. Small-Scale License Plate Detection in Dynamic Scenarios

In mobile inspection and road perception scenarios, license plates may appear as relatively small or distant targets due to non-fixed viewpoints and varying acquisition distances. Their texture and edge information may be weakened during repeated downsampling, leading to missed detections and inaccurate localization [20]. In addition, images captured by mobile robots are commonly affected by viewpoint variation, motion blur, complex background interference, and partial occlusion, which further increases the difficulty of license plate detection [5]. Compared with general object detection, license plate detection not only requires accurate bounding-box localization, but also needs to provide stable candidate regions for subsequent character recognition.

To address small-object detection challenges, existing studies have explored multi-scale feature fusion, feature pyramid enhancement, detailed spatial feature preservation, and attention mechanisms to strengthen small-target representation [21]. For example, Ma et al. proposed ISOD in *The Visual Computer*, which improves small-object detection by constructing an extended scale feature pyramid network and strengthening multi-scale feature representation [22]. These methods show that preserving detailed spatial information and enhancing multi-scale features are beneficial for small-object perception. However, additional feature enhancement operations may also increase parameters and computational overhead, which is not always suitable for real-time deployment on resource-constrained robotic platforms.

Contextual modeling is another important strategy for improving detection robustness in complex scenes. Existing studies have shown that attention mechanisms can help enhance discriminative feature representation and suppress background interference [23]. For distant license plates, surrounding contextual information can help distinguish true license plate regions from visually similar background patterns, such as traffic signs, vehicle grilles, and reflective areas. Based on these considerations, the proposed YOLOv8-MGL introduces LSKA-based contextual enhancement to compensate for the representation loss caused by lightweight feature aggregation. Meanwhile, Ghost-based lightweight modules are used to reduce redundant computation in the feature aggregation process, enabling the detector to maintain reliable small-license-plate detection performance under CPU-oriented edge deployment constraints.

### 2.3. Lightweight License Plate Recognition Methods

Traditional license plate recognition methods usually rely on sequential procedures such as image preprocessing, license plate localization, character segmentation, and character classification. Although these methods are relatively interpretable and computationally efficient in constrained environments, they are sensitive to illumination variation, perspective distortion, character adhesion, contamination, occlusion, and image blur. With the development of deep learning, sequence modeling-based license plate recognition methods have gradually become mainstream. CRNN performs character sequence recognition by combining convolutional and recurrent neural networks [24,25], while LPRNet adopts a segmentation-free lightweight recognition framework and directly maps license plate images to character sequences [26]. Owing to its compact structure and efficient inference process, LPRNet is suitable for edge deployment scenarios.

However, under the dynamic acquisition conditions of mobile robots, motion blur, low resolution, and perspective variation can weaken fine-grained character features and reduce recognition accuracy. In particular, character strokes may become indistinct, local edges may be degraded, and visually similar characters are more likely to be confused. To improve recognition robustness, attention mechanisms and feature enhancement strategies have been introduced into recognition networks. Nevertheless, many parameterized attention modules increase model complexity, which may conflict with the deployment requirements of CPU-only mobile edge platforms.

Therefore, for mobile robot license plate recognition, a practical strategy is to enhance critical character features with minimal additional computational cost. SimAM [27] estimates neuron importance through an energy function and performs parameter-free feature recalibration, which is consistent with the lightweight deployment requirement of edge-oriented recognition networks. Motivated by this property, this paper embeds SimAM into LPRNet to strengthen discriminative character responses under motion blur, perspective distortion, and local low-resolution degradation while preserving the lightweight nature of the recognition model.

In summary, existing studies have made progress in lightweight object detection, small-object perception, and license plate recognition. However, license plate detection and recognition for mobile robotic edge platforms still face the challenge of jointly balancing detection accuracy, recognition robustness, and CPU-oriented inference efficiency. To address this problem, this paper proposes a lightweight detection-and-recognition framework for mobile robot license plate perception. The proposed framework integrates Ghost-based computational redundancy reduction, LSKA-based contextual compensation, and SimAM-based character feature recalibration, thereby improving practical deployment capability under resource-constrained dynamic scenarios.

## 3. Methodology

### 3.1. Overall System Framework

**Figure 1:**
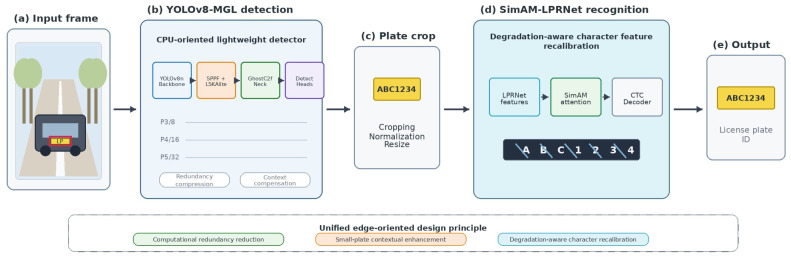
Overall architecture of the proposed license plate detection-and-recognition framework.

To address the practical requirements of license plate perception for mobile inspection robots under CPU-oriented edge deployment conditions, this paper proposes a lightweight decoupled detection-and-recognition framework, as shown in Figure 1. The proposed framework consists of two sequential stages: license plate detection and license plate recognition. In the detection stage, the input image is first processed by the proposed YOLOv8-MGL detector to localize license plate regions. The detected license plate regions are then cropped and fed into the recognition module, where SimAM-LPRNet performs segmentation-free character sequence recognition without explicit character segmentation.

The framework is designed according to the visual perception characteristics of mobile robotic inspection scenarios. Compared with fixed-camera license plate recognition, mobile robots usually operate under limited computational resources, long-distance imaging, continuous motion, and complex background interference. These factors may cause license plates to appear at relatively small scales and may introduce motion blur, perspective distortion, and local degradation, which affect both bounding-box localization and character recognition. Therefore, the proposed framework follows a coordinated design principle that combines lightweight computation, scale-aware feature enhancement, and degradation-aware character recalibration.

Specifically, in the detection stage, YOLOv8-MGL is developed based on YOLOv8n to reduce redundant convolutional computation while enhancing contextual representation for scale-varying and degraded license plate regions. GhostC2f-based lightweight feature aggregation is adopted to improve computational efficiency, and LSKAlite-based contextual enhancement is introduced to strengthen the representation of license plate regions in complex backgrounds. In the recognition stage, SimAM is embedded into LPRNet to enhance discriminative character responses under motion blur, local low resolution, and perspective variation without introducing additional learnable parameters. Through this decoupled yet coordinated design, the proposed framework aims to achieve a balance among detection accuracy, recognition accuracy, model complexity, and CPU-oriented inference efficiency in mobile robotic scenarios.

### 3.2. Lightweight Detection Network Design for CPU-Oriented Edge Deployment

**Figure 2:**
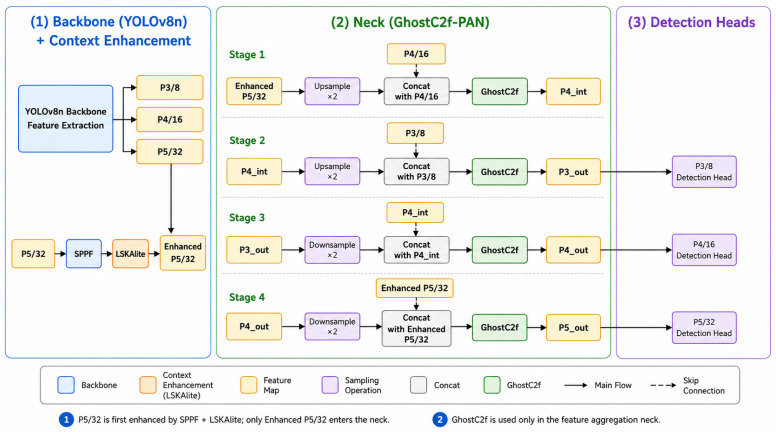
Architecture of the proposed YOLOv8-MGL detection network.

To meet the practical license plate detection requirement of mobile inspection robots on CPU-oriented edge platforms, this paper develops a lightweight detection network, termed YOLOv8-MGL, based on YOLOv8n. The overall architecture of the proposed network is shown in Figure 2. The design objective of YOLOv8-MGL is not merely to reduce model size, but to achieve a balanced trade-off among lightweight inference, contextual representation, and license plate localization under scale-varying and dynamic robot-view imaging conditions.

In mobile robotic inspection scenarios, license plate detection is affected by several practical constraints. First, the deployment platform often has limited computing resources, and the detector should operate without relying on a high-performance GPU. Second, license plates captured from a moving robot may appear at relatively small scales, especially under long-distance or non-fixed-view imaging conditions, and their spatial details can be weakened by repeated downsampling. Third, dynamic image acquisition introduces motion blur, viewpoint variation, illumination changes, and complex background interference, which further increases the difficulty of robust localization.

To address these challenges, YOLOv8-MGL follows a coordinated design principle of *redundancy compression and contextual compensation*. Specifically, the YOLOv8n backbone is retained to extract multi-scale feature maps, including P3/8, P4/16, and P5/32. The high-level P5/32 feature is first processed by the SPPF module and then enhanced by LSKAlite to obtain an enhanced P5/32 representation with stronger contextual information. This enhanced high-level feature is subsequently fed into the GhostC2f-PAN neck for multi-scale feature aggregation.

In the neck, GhostC2f modules are introduced to replace the original C2f modules in the feature aggregation process, thereby reducing redundant convolutional computation while preserving the multi-scale fusion capability of the PAN structure. Following the top-down path, the enhanced P5/32 feature is upsampled and concatenated with P4/16 to generate an intermediate P4 feature, denoted as P4_int. Then, P4_int is further upsampled and concatenated with P3/8 to obtain P3_out for small-scale prediction. In the bottom-up path, P3_out is downsampled and concatenated with P4_int to produce P4_out, while P4_out is further downsampled and concatenated with the enhanced P5/32 feature to produce P5_out. Finally, P3_out, P4_out, and P5_out are fed into three detection heads corresponding to P3/8, P4/16, and P5/32, respectively.

Through this design, GhostC2f mainly contributes to lightweight feature aggregation, while LSKAlite provides contextual compensation for scale-varying and degraded license plate regions after the SPPF module. The proposed YOLOv8-MGL therefore reduces model complexity while maintaining discriminative multi-scale feature representation for CPU-oriented edge deployment.

#### 3.2.1. GhostC2f-Based Lightweight Feature Aggregation

The neck network of YOLOv8 is responsible for fusing multi-scale features from different semantic levels. Although the original C2f module provides effective gradient flow and feature aggregation, repeated convolutional transformations in the neck may still introduce redundant computation, especially when the detector is deployed on CPU-only edge devices. Therefore, this paper designs a GhostC2f module to replace the original C2f modules in the feature aggregation neck.

**Figure 3:**
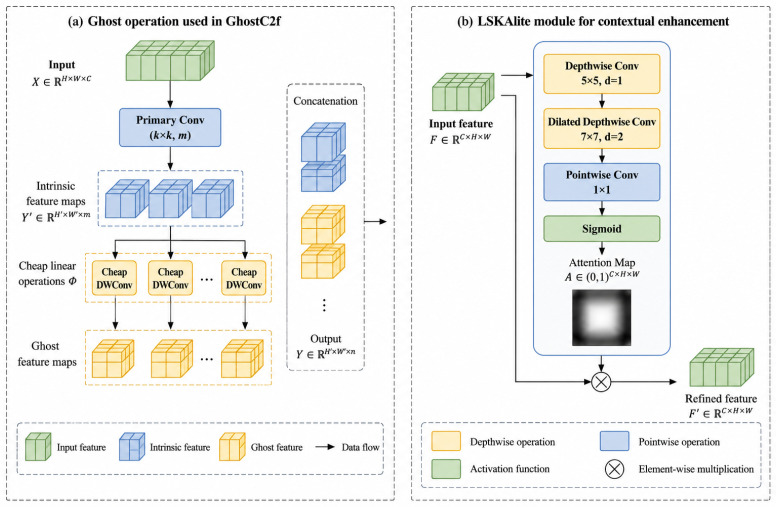
Schematic illustration of the lightweight enhancement modules used in YOLOv8-MGL. (**a**) Ghost operation used in the GhostC2f module. (**b**) Structure of the proposed LSKAlite module for contextual enhancement.

The lightweight enhancement modules used in YOLOv8-MGL are illustrated in Figure 3. Figure 3a shows the Ghost operation used in GhostC2f, while Figure 3b shows the structure of the proposed LSKAlite module.

The Ghost operation is based on the observation that many feature maps generated by standard convolution contain redundant information. Instead of generating all output feature maps using dense convolution, the Ghost operation first produces a smaller number of intrinsic feature maps and then generates additional Ghost feature maps through low-cost linear transformations.

Given an input feature map $X\in R^{H\times W\times C}$, the primary convolution generates intrinsic feature maps $Y^{\prime }$ as

(1) $$Y^{\prime }=X*f_{\mathrm{primary}}+b,$$

where $f_{\mathrm{primary}}\in R^{k\times k\times C\times m}$ denotes the primary convolution kernel, *m* is the number of intrinsic feature maps, * denotes the convolution operation, and *b* is the bias term. Since *m* is smaller than the target output channel number, this step reduces the computational cost of dense convolution.

Then, cheap linear transformations are applied to the intrinsic feature maps to generate additional Ghost feature maps. For the *i*-th intrinsic feature map $y_{i}^{\prime }$, the generated feature maps can be formulated as

(2) $$y_{ij}=\Phi_{i,j}\left(y_{i}^{\prime }\right),\;i=1,\ldots ,m,\;j=1,\ldots ,s,$$

where $\Phi_{i,j}\left(\cdot \right)$ denotes the *j*-th cheap transformation applied to $y_{i}^{\prime }$, and *s* represents the transformation ratio. In this work, depthwise convolution is used as the cheap transformation. The intrinsic feature maps and the generated Ghost feature maps are concatenated to form the final output.

Based on this operation, GhostC2f preserves the split-and-concatenate feature aggregation manner of C2f while replacing the intermediate standard bottleneck transformations with lightweight Ghost bottlenecks. Since the neck network performs repeated feature fusion across different scales, applying GhostC2f in this stage can effectively reduce redundant feature transformation without altering the overall detection pipeline. This design provides the lightweight computational foundation of YOLOv8-MGL.

#### 3.2.2. Contextual Enhancement Based on LSKAlite

Although GhostC2f reduces redundant computation, excessive lightweight compression may weaken contextual representation, especially for scale-varying and degraded license plate regions in dynamic robotic scenes. In some robot-view images, license plates may appear at relatively small scales or under degraded visual conditions, and they may be confused with visually similar background patterns, such as vehicle grilles, traffic signs, and reflective regions. Therefore, an efficient contextual compensation mechanism is required to enhance feature discrimination while maintaining low computational overhead.

To this end, this paper introduces LSKAlite after the SPPF module. The SPPF module aggregates high-level semantic information through fast spatial pyramid pooling, while LSKAlite further enhances long-range contextual perception before the features are decoded by the neck network. Unlike standard large-kernel convolution, LSKAlite adopts depthwise and dilated depthwise convolution operations to enlarge the effective receptive field with limited parameter and computational cost, making it suitable for CPU-oriented edge deployment.

As shown in Figure 3b, given an input feature map $F\in R^{C\times H\times W}$, LSKAlite first applies a depthwise convolution to capture local spatial dependencies. Then, a dilated depthwise convolution is used to enlarge the receptive field and strengthen long-range spatial modeling. Finally, a pointwise convolution and sigmoid activation are used to generate an attention map for feature recalibration. The overall process can be expressed as

(3) $$F^{\prime }=F⊗\sigma \left({\mathrm{Conv}}_{1\times 1}\left({\mathrm{DWConv}}_{d}\left(\mathrm{DWConv}(F)\right)\right)\right),$$

where $F^{\prime }$ denotes the refined feature map, DWConv(·) denotes depthwise convolution, ${\mathrm{DWConv}}_{d}\left(\cdot \right)$ denotes dilated depthwise convolution, ${\mathrm{Conv}}_{1\times 1}\left(\cdot \right)$ denotes pointwise convolution, σ(·) represents the sigmoid activation function, and ⊗ denotes element-wise multiplication.

By inserting LSKAlite after SPPF, the detector can enhance high-level contextual cues before multi-scale feature fusion. This design compensates for the potential representation loss caused by lightweight feature aggregation and improves the ability of the network to distinguish scale-varying and degraded license plate regions from complex backgrounds.

#### 3.2.3. Collaborative Lightweight Detection Design

The proposed YOLOv8-MGL combines GhostC2f and LSKAlite in a complementary manner. GhostC2f focuses on reducing redundant computation in the feature aggregation neck, while LSKAlite provides contextual compensation for scale-varying and degraded license plate regions. Therefore, the proposed detector is not a simple stacking of independent modules, but a coordinated lightweight design that considers both computational efficiency and feature representation.

From the perspective of edge deployment, this design reduces the parameter footprint and theoretical computational cost of the detector while maintaining effective multi-scale feature fusion and contextual modeling. Compared with the original YOLOv8n, YOLOv8-MGL achieves a more compact detection structure and maintains practical CPU-oriented inference capability. The detailed comparisons of detection accuracy, model complexity, and CPU inference speed are presented in Section 4.

### 3.3. Lightweight License Plate Recognition Based on SimAM-LPRNet

After the license plate region is localized by the proposed YOLOv8-MGL detector, the cropped plate image is further fed into the recognition module to generate the corresponding character sequence. In mobile robotic inspection scenarios, license plate recognition is different from recognition under fixed-camera conditions. Due to robot motion, mechanical vibration, viewpoint changes, and complex illumination, the cropped license plate regions may suffer from motion blur, perspective distortion, local low resolution, and uneven brightness. These degradations weaken fine-grained character strokes and increase the confusion between visually similar characters, such as “0” and “O”, “1” and “I”, or “8” and “B”. Therefore, the recognition module should not only maintain lightweight inference capability, but also enhance discriminative character responses under dynamic visual degradation.

In this paper, LPRNet is adopted as the basic recognition network because it follows a segmentation-free sequence recognition paradigm. Compared with traditional license plate recognition methods that rely on character segmentation, LPRNet directly extracts sequence features from the cropped license plate image and predicts the final character sequence through a connectionist temporal classification strategy. This structure avoids the error accumulation caused by character segmentation and is suitable for edge-oriented deployment. However, the original LPRNet mainly relies on convolutional feature extraction and lacks an explicit feature recalibration mechanism for degraded character regions. When the license plate image is blurred or locally low-resolution, important character strokes may be weakened during feature extraction, which affects the final recognition accuracy.

**Figure 4:**
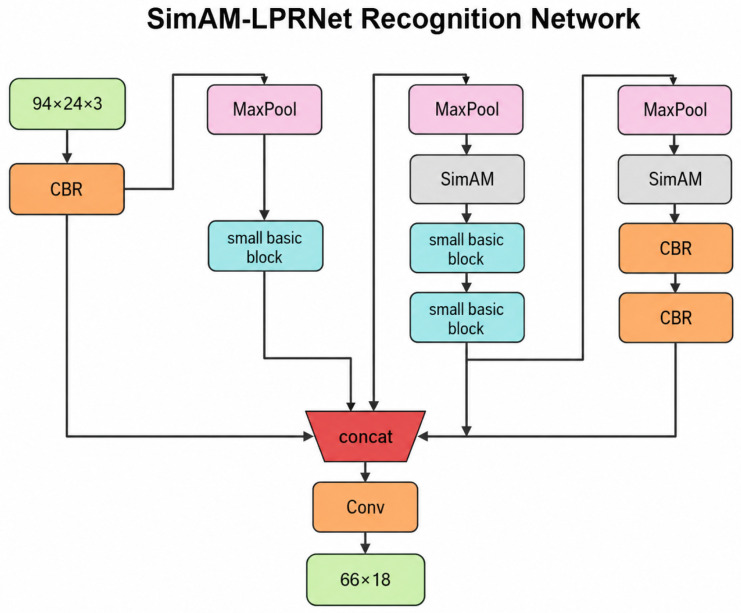
Architecture of the proposed SimAM-LPRNet recognition network. SimAM is inserted after the last two deeper MaxPool layers in the feature extraction stage to enhance discriminative character responses without introducing additional learnable parameters.

To address this problem, this paper introduces the parameter-free SimAM attention mechanism into the feature extraction stage of LPRNet, forming the proposed SimAM-LPRNet recognition network. The improved recognition architecture is shown in Figure 4. The key idea is to enhance important character-related neurons while suppressing less informative background responses without introducing additional learnable parameters. This design is consistent with the overall lightweight principle of the proposed framework: the detection stage reduces redundant computation through GhostC2f and compensates contextual representation through LSKAlite, while the recognition stage further recalibrates degraded character features through SimAM.

Different from channel attention mechanisms that depend on global channel descriptors and fully connected layers, or spatial attention mechanisms that require additional convolutional operations, SimAM estimates the importance of each neuron through an analytically defined energy function. In other words, SimAM does not explicitly learn additional attention parameters. Instead, it measures whether a neuron is sufficiently distinguishable from its surrounding neurons. A neuron with stronger separability is regarded as more informative and is assigned a larger response weight. This property makes SimAM suitable for lightweight license plate recognition, especially when the model is deployed on CPU-only edge platforms.

Given an input feature map *X*, SimAM evaluates the importance of each neuron by constructing an energy function based on the difference between the target neuron and other neurons in the same channel. The closed-form solution of the energy function can be expressed as

(4) $$e_{t}^{*}=\frac{4(\sigma^{2}+\lambda )}{{\left(t-\mu \right)}^{2}+2\sigma^{2}+2\lambda },$$

where $e_{t}^{*}$ denotes the minimum energy of the *t*-th neuron, μ and $\sigma^{2}$ represent the mean and variance computed from all spatial neurons within the corresponding channel, and λ is a regularization coefficient. A lower energy value indicates that the neuron is more discriminative with respect to its surrounding responses. Therefore, the reciprocal of the energy value can be used to generate the attention weight for feature recalibration.

Based on the above energy measurement, the recalibrated feature map can be formulated as

(5) $$X^{\prime }=\mathrm{sigmoid}\left(\frac{1}{E}\right)⊙X,$$

where *X* and $X^{\prime }$ denote the input and recalibrated feature maps, respectively, *E* represents the energy measurement of neurons, sigmoid(·) is used to normalize the attention response, and ⊙ denotes element-wise multiplication.

By embedding SimAM into LPRNet, the recognition network can strengthen character-sensitive regions such as strokes, edges, and local structural details, while suppressing redundant or degraded background responses in the cropped plate image. Since SimAM introduces no additional learnable parameters, the improved recognition network maintains the lightweight property of LPRNet while improving its robustness to motion blur, perspective distortion, and local low-resolution degradation. In this way, SimAM-LPRNet complements the YOLOv8-MGL detection module and forms a complete lightweight license plate perception framework for mobile robotic edge deployment.

### 3.4. Training Strategy and Loss Function

The proposed framework consists of two sequential stages: license plate detection and license plate recognition. Therefore, the training objectives are designed according to the functional characteristics of the two modules. The YOLOv8-MGL detector is trained to accurately localize license plate regions under scale-varying and dynamic-background conditions, while the SimAM-LPRNet recognizer is trained to predict the corresponding character sequence from the cropped license plate image. Since the two stages solve different tasks, this paper adopts task-specific optimization objectives for detection and recognition, respectively.

For the detection stage, this paper follows the task-aligned learning strategy adopted in YOLOv8. In mobile robotic inspection scenarios, license plates may appear at varying scales, and their visual responses may be weakened by motion blur, viewpoint variation, and complex background interference. Under such conditions, it is important to improve the consistency between classification confidence and localization quality during positive sample assignment. Task-aligned learning considers both classification prediction and localization quality when selecting positive samples, which helps the detector assign more reliable supervision signals to license plate regions under scale variation and visual degradation. This strategy can reduce mismatches between high classification confidence and inaccurate bounding box localization, especially under long-distance or degraded imaging conditions. The overall detection loss of YOLOv8-MGL can be formulated as

(6) $$L_{\det}=\lambda_{\mathrm{cls}}L_{\mathrm{cls}}+\lambda_{\mathrm{box}}L_{\mathrm{box}}+\lambda_{\mathrm{dfl}}L_{\mathrm{DFL}},$$

where $L_{\mathrm{cls}}$ denotes the classification loss, $L_{\mathrm{box}}$ denotes the bounding box regression loss, and $L_{\mathrm{DFL}}$ denotes the distribution focal loss. The coefficients $\lambda_{\mathrm{cls}}$, $\lambda_{\mathrm{box}}$, and $\lambda_{\mathrm{dfl}}$ are used to balance the contributions of classification, localization, and bounding box distribution learning. Specifically, the classification loss helps the network distinguish license plate regions from complex background patterns, while the bounding box regression loss optimizes the spatial localization of license plate regions. The distribution focal loss further refines the boundary distribution representation, which is beneficial for improving localization precision when license plates are affected by scale variation, blur, or partial degradation.

For the recognition stage, the cropped license plate image is regarded as an ordered character sequence. Since license plate characters are arranged horizontally and the exact alignment between image features and character labels is difficult to determine manually, this paper adopts the connectionist temporal classification loss to train the SimAM-LPRNet recognition network. This loss function enables sequence prediction without requiring character-level segmentation annotations, which is consistent with the segmentation-free design of LPRNet.

Given an input license plate image *I*, the recognition network outputs a probability sequence $P=\{p_{1},p_{2},\ldots ,p_{T}\}$ over the predefined character set, where *T* denotes the length of the output feature sequence. Let $Y=\{y_{1},y_{2},\ldots ,y_{L}\}$ denote the ground-truth license plate character sequence. The recognition loss can be expressed as

(7) $$L_{\mathrm{rec}}=-\log P\left(Y|I\right),$$

where P(Y|I) denotes the probability of predicting the correct character sequence from the input license plate image. During training, all possible alignments between the network output sequence and the target character sequence are considered, allowing the model to learn character recognition without explicit character segmentation.

Through the above task-specific optimization strategy, the detection and recognition modules are trained according to their respective objectives. The detection loss improves the localization accuracy of license plate regions under scale variation and complex-background conditions, while the recognition loss enhances sequence-level character prediction from cropped license plates. Meanwhile, the lightweight structures introduced in YOLOv8-MGL and the parameter-free SimAM mechanism in LPRNet do not change the basic training objective, which helps maintain training stability while improving the balance between accuracy and efficiency. As a result, the proposed framework can achieve reliable license plate perception under CPU-oriented mobile robotic edge deployment conditions.

## 4. Experimental Validation

### 4.1. Dataset and Experimental Settings

**Figure 5:**
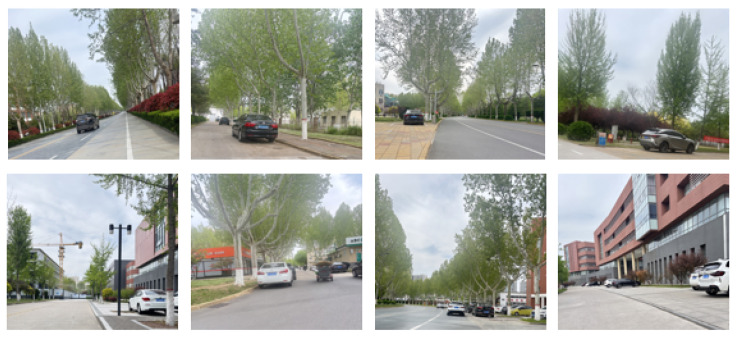
Representative samples from the self-built EDRV-LP dataset collected under mobile inspection and road-view scenarios.

To evaluate the proposed lightweight license plate detection and recognition framework, experiments were conducted on the self-built EDRV-LP dataset. In this work, EDRV-LP denotes the Extreme Dynamic Robot-View License Plate dataset. The dataset was collected from mobile inspection and road-view scenarios, where images were captured under non-fixed viewpoints and complex outdoor backgrounds. Different from license plate images collected by fixed surveillance cameras, the samples in EDRV-LP contain practical visual degradations commonly encountered in mobile robotic inspection, including small-scale or distant license plate appearance, viewpoint variation, illumination changes, motion blur, and background interference. Several representative samples of the dataset are shown in Figure 5.

The EDRV-LP dataset contains 2000 images in total. For the detection task, all images were annotated in YOLO format, where each image corresponds to one label file containing the normalized license plate bounding box. To avoid evaluating the final model only on the validation set, the dataset was divided into 1600 training images, 200 validation images, and 200 held-out test images. The validation set was used for early stopping and model selection, while the held-out test set was used only for final performance reporting. All compared detection models were trained using the same training split and evaluated under the same validation and test protocols to ensure a fair comparison.

To provide a more objective description of the dataset difficulty, automatic image-quality proxy statistics were computed based on the annotated license plate regions. Specifically, the relative bounding-box area was used to describe license plate scale, the mean brightness of the cropped license plate region was used as a low-light proxy, and the Laplacian variance of the cropped license plate region was used as a blur or low-texture proxy. A sample was regarded as a small-plate sample when the license plate bounding-box area was lower than 1% of the image area, as a low-light sample when the mean plate-region brightness was lower than 80, and as a blurred or low-texture sample when the Laplacian variance was lower than 100. These statistics are automatic proxy indicators rather than manual semantic labels. According to this analysis, the median license plate area ratios of the training, validation, test, and overall sets were 2.594%, 3.022%, 1.548%, and 2.494%, respectively. In the overall dataset, 26 images were identified as small-plate samples, 295 images were identified as low-light samples, and 87 images were identified as blurred or low-texture samples, corresponding to 1.3%, 14.8%, and 4.3% of the dataset, respectively. Although the strict small-plate proxy accounts for a limited proportion of the overall dataset, the held-out test set has a smaller median plate area ratio than the training and validation sets, indicating that the final evaluation includes more challenging scale conditions. Therefore, the dataset is described as containing small-scale and distant license plate cases rather than being dominated by tiny targets.

For the recognition task, a cropped-license-plate recognition subset was constructed from the ground-truth bounding boxes of the EDRV-LP dataset. The training, validation, and test splits of the recognition subset were kept consistent with those of the detection dataset, resulting in 1600 training crops, 200 validation crops, and 200 held-out test crops. The corresponding license plate character strings were obtained from the filename-based annotation format used in this dataset, which follows the CCPD-style naming convention, and were used as sequence labels. The recognition subset contains both 7-character conventional license plates and 8-character extended license plates. Specifically, the training set contains 425 7-character plates and 1175 8-character plates, the validation set contains 43 7-character plates and 157 8-character plates, and the held-out test set contains 167 7-character plates and 33 8-character plates. Overall, the recognition subset contains 635 7-character plates and 1365 8-character plates. The character set contains 43 categories, including digits, uppercase letters, and Chinese province abbreviations appearing in the dataset.

In the recognition experiments, ground-truth crops were used to train and evaluate the recognition models, so that the influence of the recognition network design could be analyzed independently from detection localization errors. The SimAM-LPRNet experiments were conducted under this ground-truth crop setting to evaluate the proposed recognition module at the cropped-plate level. For supplementary system-level evaluation, the original evaluation images were first processed by YOLOv8-MGL, and the detected license plate regions were then cropped and fed into an additional CRNN-CTC recognizer for variable-length 7-character and 8-character plate sequence recognition. This additional recognizer was used only for the supplementary end-to-end pipeline and does not change the proposed YOLOv8-MGL detection architecture or the SimAM-LPRNet recognition ablation. A prediction was counted as correct only when the complete predicted license plate string exactly matched the ground-truth sequence.

To further evaluate external generalization, a non-overlapping external CCPD subset containing 200 license plate images was additionally constructed for external validation. The images in this subset were not used for training, validation, hyperparameter tuning, or checkpoint selection. License plate bounding boxes and character strings were parsed from the CCPD filenames. Exact file-hash checking was performed between this external CCPD subset and EDRV-LP, and no duplicated images were found. Therefore, this subset was used only as an external test set to evaluate the generalization ability of YOLOv8-MGL and the supplementary YOLOv8-MGL + CRNN-CTC pipeline.

Ethical and privacy considerations were also taken into account during dataset construction and evaluation. The collected images were used only for academic research on license plate detection and recognition. No personal identity attributes such as driver identity, pedestrian identity, or owner information were annotated. The annotations were limited to license plate bounding boxes and character strings required for algorithm evaluation. The dataset is not publicly released in this study, and access can be provided by the corresponding author upon reasonable request for research purposes.

For the detection experiments, all models were trained with an input resolution of 640×640. The main training hyperparameters were kept consistent across different models. Stochastic gradient descent (SGD) was used as the optimizer, with an initial learning rate of 0.01, momentum of 0.9, and weight decay of 0.005. The batch size was set to 64, and the maximum number of training epochs was set to 300. Early stopping was enabled with a patience of 100 epochs to avoid unnecessary training when the validation performance no longer improved. To reduce overfitting on the relatively small dataset, standard YOLO data augmentation strategies were adopted during detection training, including color perturbation, random scaling, random translation, horizontal flipping, and mosaic augmentation.

The recognition models were trained using cropped license plate images resized according to their respective network input settings. For the CRNN-CTC module used in the supplementary end-to-end validation, the input crop size was set to 192×48. Connectionist temporal classification (CTC) loss was adopted for sequence prediction without requiring character-level segmentation annotations. The best recognition checkpoint was selected according to validation accuracy, and the held-out test set was used only for final evaluation.

The detection models were trained on an NVIDIA RTX 3090 GPU. Since the target application of this work is CPU-oriented robotic edge perception, model complexity and CPU-only inference efficiency were also evaluated. The number of parameters, GFLOPs, average inference latency, and FPS were reported to analyze deployment suitability. CPU-only inference was measured with batch size 1 after disabling GPU acceleration. It should be noted that the CPU-only experiment was conducted on an Intel Xeon Platinum 8260C CPU @ 2.30 GHz, which is a server-class CPU rather than a low-power embedded robotic processor. Therefore, the CPU-only evaluation is used to demonstrate CPU-oriented inference feasibility, while further validation on practical embedded robotic hardware remains future work.

### 4.2. Evaluation Metrics

To quantitatively evaluate the proposed framework, different metrics were adopted for the detection and recognition tasks. For the license plate detection task, precision, recall, mAP_50_, mAP_50:95_, the number of parameters, and GFLOPs were used as evaluation metrics. For the license plate recognition task, recognition accuracy and final training loss were used to measure the character sequence recognition performance.

Precision and recall are used to evaluate the correctness and completeness of license plate localization. Precision measures the proportion of correctly detected license plates among all predicted license plate regions, while recall measures the proportion of correctly detected license plates among all ground-truth license plates. They are defined as follows:

(8) $$\mathrm{Precision}=\frac{TP}{TP+FP}$$

(9) $$\mathrm{Recall}=\frac{TP}{TP+FN}$$

where TP, FP, and FN denote true positives, false positives, and false negatives, respectively.

The mean average precision at an IoU threshold of 0.5, denoted as mAP_50_, was adopted to evaluate the overall detection accuracy. In addition, mAP_50:95_ was used to evaluate localization quality under stricter IoU thresholds. Compared with mAP_50_, mAP_50:95_ is more sensitive to bounding box localization accuracy and can provide a more comprehensive evaluation of detection performance. Since the mAP_50_ values of lightweight detectors tend to be close on the held-out EDRV-LP test set, mAP_50:95_ is particularly useful for distinguishing their localization quality.

In addition to detection accuracy, model complexity was evaluated using the number of parameters and GFLOPs. The number of parameters reflects the model storage cost, while GFLOPs reflects the computational cost of a forward inference process. These two metrics are important for evaluating the deployment suitability of lightweight detection models on resource-constrained edge platforms.

For the license plate recognition task, recognition accuracy was used as the primary evaluation metric. A license plate is regarded as correctly recognized only when the predicted character sequence is completely consistent with the ground-truth sequence. Therefore, this metric reflects the complete-sequence recognition capability of the recognition network at the cropped-plate level. The recognition accuracy is defined as

(10) $$\mathrm{Accuracy}=\frac{N_{\mathrm{correct}}}{N_{\mathrm{total}}}\times 100\%$$

where $N_{\mathrm{correct}}$ denotes the number of correctly recognized license plates, and $N_{\mathrm{total}}$ denotes the total number of evaluated license plates.

The final training loss was also reported to reflect the convergence behavior of different recognition models. However, since a lower loss does not necessarily indicate better sequence-level recognition accuracy, recognition accuracy was used as the main criterion for comparing different LPRNet variants.

### 4.3. Detection Performance Comparison

To evaluate the effectiveness of the proposed YOLOv8-MGL detector under a more reliable experimental protocol, the final detection performance was reported on the held-out EDRV-LP test set. The validation set was used only for early stopping and model selection, while the held-out test set was not involved in training or hyperparameter tuning. Since YOLOv8-MGL is developed based on YOLOv8n, YOLOv8n was used as the primary baseline for evaluating the influence of the proposed lightweight and contextual enhancement design. To broaden the comparison with representative lightweight and larger-scale detectors, YOLOv5n-u, YOLOv10n, and YOLOv8s were also included under the same training and held-out test protocol. The comparison mainly considers detection accuracy and model complexity, including precision, recall, mAP_50_, mAP_50:95_, the number of parameters, and GFLOPs. The results are shown in Table 1.

As shown in Table 1, all compared detectors achieve high mAP_50_ values on the held-out EDRV-LP test set, indicating that the single-class license plate detection task is close to saturation at the IoU threshold of 0.5. Therefore, mAP_50:95_, model complexity, and CPU-oriented inference efficiency are more informative for evaluating the practical trade-off among different models.

Compared with the YOLOv8n baseline, YOLOv8-MGL reduces the number of parameters from 3.01 M to 2.77 M and decreases the computational cost from 8.10 to 7.50 GFLOPs. Meanwhile, mAP_50:95_ increases from 0.687 to 0.711, while mAP_50_ remains 0.995. These results indicate that the proposed GhostC2f- and LSKAlite-based design improves stricter localization quality while reducing model complexity.

It can also be observed that YOLOv8s achieves the highest mAP_50:95_ among the compared models. However, it requires 11.14 M parameters and 28.65 GFLOPs, which are substantially larger than those of YOLOv8-MGL. YOLOv5n-u and YOLOv10n obtain comparable mAP_50:95_ values, but their CPU-only inference efficiency is lower than that of YOLOv8-MGL, as further analyzed in Section 4.6. Therefore, YOLOv8-MGL is not intended to outperform all comparison models in every single metric. Instead, it provides a favorable trade-off among compact model size, reliable localization quality, and CPU-oriented inference feasibility for mobile robotic license plate perception.

### 4.4. Ablation Study of YOLOv8-MGL

To analyze the contribution of each component in the proposed detection network, an ablation study was conducted on the held-out EDRV-LP test set. YOLOv8n was used as the baseline model. Based on this baseline, LSKAlite and GhostC2f were introduced separately, and the complete YOLOv8-MGL model was evaluated by integrating both components. The ablation results are shown in Table 2.

As shown in Table 2, all model variants achieve a mAP_50_ of 0.995 on the held-out test set. This indicates that the single-class license plate detection task is close to saturation at the IoU threshold of 0.5. Therefore, mAP_50:95_ is more informative for evaluating localization quality under stricter IoU thresholds.

When LSKAlite is introduced alone after the SPPF module, the number of parameters slightly increases from 3.01M to 3.09M, and the computational cost increases from 8.1 to 8.2 GFLOPs. This is because LSKAlite introduces additional contextual modeling operations. Compared with the YOLOv8n baseline, the LSKAlite-only variant improves mAP_50:95_ from 0.687 to 0.710 while maintaining the same mAP_50_ of 0.995. This result indicates that LSKAlite can enhance high-level contextual representation and improve localization quality for license plate regions, although it is not designed as a model-compression module.

When GhostC2f is introduced into the feature aggregation neck, the number of parameters decreases from 3.01M to 2.68M, and the computational cost decreases from 8.1 to 7.4 GFLOPs. Meanwhile, the model maintains the same mAP_50_ of 0.995 and improves mAP_50:95_ from 0.687 to 0.701. This result suggests that GhostC2f effectively reduces redundant convolutional computation in the neck while preserving reliable detection performance. Therefore, GhostC2f is the main component responsible for the lightweight design of YOLOv8-MGL.

For the complete YOLOv8-MGL model, GhostC2f and LSKAlite are jointly integrated. Compared with the YOLOv8n baseline, YOLOv8-MGL reduces the parameter count from 3.01 M to 2.77 M and decreases the computational cost from 8.1 to 7.5 GFLOPs, while maintaining the same mAP_50_ of 0.995. In addition, mAP_50:95_ increases from 0.687 to 0.711. This result supports the complementary relationship between GhostC2f and LSKAlite: GhostC2f reduces redundant computation in the feature aggregation neck, while LSKAlite helps compensate for contextual representation loss caused by lightweight feature aggregation.

Overall, the ablation results indicate that the proposed YOLOv8-MGL mainly improves the trade-off between model complexity and localization quality rather than producing a large absolute gain in mAP_50_. GhostC2f contributes to redundancy reduction and model lightweighting in the feature aggregation neck, whereas LSKAlite provides contextual compensation for scale-varying and degraded license plate regions after the SPPF module. With the coordinated design of these two components, YOLOv8-MGL reduces model complexity while maintaining saturated mAP_50_ and improving mAP_50:95_ on the held-out test set. This behavior is consistent with the objective of CPU-oriented mobile robotic license plate perception, where compactness, stable localization, and practical inference efficiency should be jointly considered.

### 4.5. Recognition Performance of SimAM-LPRNet

After the license plate regions are localized by the proposed detection network, the cropped plate images are further processed by the recognition module to obtain the corresponding character sequences. In mobile robotic inspection scenarios, the cropped license plate regions are often affected by motion blur, perspective distortion, local low resolution, uneven illumination, and partial character degradation. These factors may weaken fine-grained character strokes and increase the confusion between visually similar characters. Therefore, the recognition module should not only maintain lightweight inference capability, but also enhance discriminative character responses under dynamic visual degradation.

In this paper, the original LPRNet is adopted as the baseline recognition model because it follows a segmentation-free recognition paradigm and is suitable for edge-oriented deployment. To improve its representation ability for degraded license plate characters, the parameter-free SimAM attention mechanism is introduced into LPRNet. Since the effect of attention enhancement is closely related to its insertion position, four SimAM-enhanced variants are constructed by inserting SimAM into different locations of the feature extraction network. All recognition models were trained and evaluated under the same cropped-plate data configuration using ground-truth license plate crops. This module-level evaluation was designed to isolate the influence of the recognition network structure from detection localization errors. Recognition accuracy and final training loss were used as the evaluation metrics. The results are shown in Table 3.

As shown in Table 3, the recognition performance of LPRNet can be improved when SimAM is inserted into appropriate positions. Compared with the baseline LPRNet, inserting SimAM after the last MaxPool layer improves the recognition accuracy from 83.10% to 84.90%. When SimAM is inserted after the last two MaxPool layers, the recognition accuracy reaches 87.17%, achieving the best performance among all variants and improving the baseline by 4.07 percentage points. This result indicates that SimAM can strengthen discriminative character responses in the deeper feature extraction stage, which is beneficial for recognizing degraded license plate characters.

It should also be noted that adding more attention modules does not necessarily lead to better recognition accuracy. Although the variant with SimAM after each MaxPool layer obtains a lower final training loss, its recognition accuracy is lower than that of the second variant. In addition, inserting SimAM before the penultimate CBR block even decreases the recognition accuracy compared with the baseline. This phenomenon indicates that excessive or inappropriate feature recalibration may disturb the original feature distribution of LPRNet and weaken the sequence representation required for license plate recognition. Therefore, the variant with SimAM inserted after the last two MaxPool layers is selected as the final SimAM-LPRNet structure in this paper. In the implementation, these two positions correspond to the two deeper pooling stages in the LPRNet feature extraction backbone. This placement enhances higher-level character-related features while avoiding excessive recalibration in shallow low-level features.

To further verify the effectiveness of SimAM, a comparative experiment is conducted between SimAM and the classical SE attention mechanism. In this comparison, the attention module is inserted into the same effective position of LPRNet, and the experimental results are shown in Table 4.

From Table 4, LPRNet with SimAM achieves higher recognition accuracy and lower final loss than LPRNet with SE attention. Specifically, the SimAM-enhanced model reaches 87.17% recognition accuracy, while the SE-enhanced model reaches 84.95%. This result shows that SimAM is more suitable for the lightweight recognition module in the proposed framework. The reason is that SimAM estimates neuron importance through an energy function and enhances informative responses without introducing additional learnable parameters. In contrast, SE mainly models channel-wise dependencies through additional fully connected layers, which introduces extra parameter overhead and is less consistent with the lightweight deployment objective of this work.

Overall, the recognition experiments demonstrate that the proposed SimAM-LPRNet can effectively improve module-level license plate recognition performance on cropped plate images. By enhancing discriminative character regions in a parameter-free manner, SimAM-LPRNet complements the YOLOv8-MGL detection network. The system-level detection–recognition performance using detected crops is further evaluated in the end-to-end pipeline experiment.

### 4.6. CPU-Oriented Inference Efficiency Analysis

Since the proposed framework is designed for mobile robotic edge scenarios, CPU-only inference efficiency is an important criterion in addition to detection accuracy and model complexity. In practical inspection scenarios, mobile robots or inspection devices may not always be equipped with dedicated GPUs. Therefore, the detector is expected to maintain practical inference capability under CPU-only conditions while keeping a compact model structure.

To evaluate CPU-oriented deployment efficiency, YOLOv5n-u, YOLOv8n, YOLOv10n, YOLOv8s, and the proposed YOLOv8-MGL were tested under the same CPU-only inference environment. GPU acceleration was disabled during the test, and the batch size was set to 1. The input resolution was set to 640×640, and the held-out EDRV-LP test set containing 200 images was used as the inference source. The average latency and frames per second (FPS) were recorded to evaluate practical CPU inference efficiency. The results are shown in Table 5.

As shown in Table 5, YOLOv8-MGL achieves the fastest CPU-only detection speed among the compared models, with an average latency of 41.70 ms per image and 23.98 FPS. Compared with YOLOv8n, YOLOv8-MGL reduces the number of parameters from 3.01 M to 2.77 M and decreases the computational cost from 8.10 to 7.50 GFLOPs, while improving mAP_50:95_ from 0.687 to 0.711. This result indicates that the proposed GhostC2f- and LSKAlite-based design improves the balance between model compactness, localization quality, and CPU inference speed.

YOLOv5n-u achieves a comparable mAP_50:95_ of 0.712 and has slightly lower theoretical complexity than YOLOv8-MGL. However, its CPU-only latency is 45.30 ms per image, which is slower than YOLOv8-MGL. YOLOv10n also obtains a comparable mAP_50:95_ of 0.712, but its recall is lower in the detection comparison and its CPU-only speed is lower than that of YOLOv8-MGL. YOLOv8s achieves the highest mAP_50:95_ of 0.719, but it requires 11.14 M parameters and 28.65 GFLOPs, leading to a much lower CPU-only speed of 12.74 FPS.

These results show that YOLOv8-MGL is not designed to simply maximize a single accuracy metric. Instead, it aims to provide a favorable CPU-oriented trade-off among model complexity, localization quality, and practical inference efficiency. Although YOLOv8s achieves a higher mAP_50:95_ and YOLOv5n-u and YOLOv10n achieve comparable mAP_50:95_ values, YOLOv8-MGL achieves the fastest CPU-only inference speed among the compared detectors while maintaining saturated mAP_50_ and competitive mAP_50:95_ on the held-out EDRV-LP test set.

To further examine the practical efficiency of the complete detection–recognition pipeline, CPU-only end-to-end latency was also measured. It should be noted that the detector latency reported in Table 5 was measured using the isolated detection validation process, whereas the following end-to-end timing includes additional pipeline overhead, such as image handling, detector invocation, and crop preparation. Under this complete pipeline timing scope, the average detection time of YOLOv8-MGL was 48.62 ms per image, and the average recognition time of the additional CTC-based recognition module was 20.07 ms per detected crop. The complete pipeline achieved an average total latency of 75.46 ms per image, corresponding to 13.25 FPS. This result indicates that the supplementary detection–recognition pipeline can perform CPU-only license plate detection and recognition at a practical processing speed.

It should be noted that the CPU-only evaluation was conducted on an Intel Xeon Platinum 8260C CPU platform, which is a server-class CPU rather than a dedicated embedded robotic processor. Therefore, the results should be interpreted as CPU-oriented feasibility evidence rather than final deployment performance on all embedded platforms. Further validation on practical embedded robotic hardware will be considered in future work.

### 4.7. End-to-End and External Validation

Although the preceding experiments evaluate the detection and recognition modules separately, practical license plate perception requires the complete process from raw robot-view images to final license plate strings. Therefore, a supplementary end-to-end pipeline experiment was conducted to evaluate system-level behavior under detected-crop conditions. This experiment is used as an additional pipeline-level validation rather than a replacement for the SimAM-LPRNet module-level recognition evaluation. Specifically, the SimAM-LPRNet experiments in Section 4.5 evaluate cropped-plate recognition under ground-truth crop conditions, whereas this section examines whether YOLOv8-MGL can provide reliable detected crops for downstream sequence recognition.

In this experiment, the original images were first processed by YOLOv8-MGL, and the detected license plate regions were then cropped and sent to an additional CRNN-CTC recognition module for variable-length plate sequence recognition. A prediction was counted as correct only when the complete predicted license plate string exactly matched the ground-truth sequence. The end-to-end recognition results are summarized in Table 6.

In Table 6, Exact Acc. is computed over all images in each subset, while Char. Acc. is computed on the recognized plate strings from successfully detected crops. The supplementary YOLOv8-MGL + CRNN-CTC pipeline successfully detected all 200 images in the held-out EDRV-LP test set and achieved an exact plate recognition accuracy of 0.910 and a character-level accuracy of 0.981. For 7-character and 8-character license plates in the EDRV-LP test set, the exact recognition accuracies were 0.898 and 0.970, respectively. These results indicate that YOLOv8-MGL can provide reliable cropped plate regions for subsequent sequence recognition under robot-view imaging conditions.

**Figure 6:**
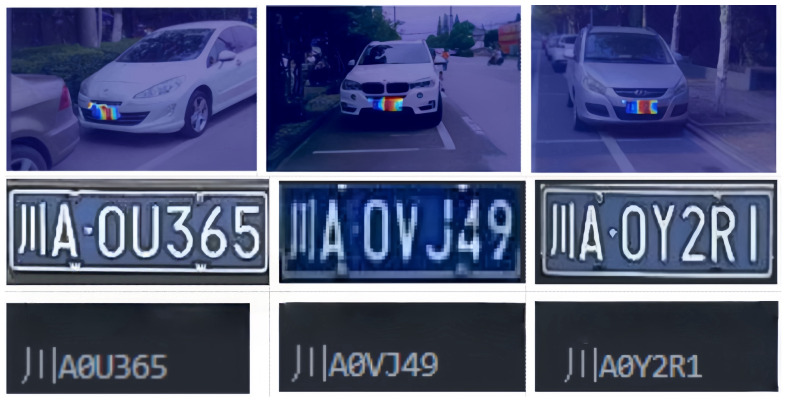
Qualitative examples of the supplementary end-to-end pipeline. The first row shows original robot-view input images, the second row shows detected license plate crops, and the third row shows the corresponding recognition results. The province abbreviation in the recognition results denotes Sichuan Province.

Several qualitative examples of the supplementary end-to-end pipeline are shown in Figure 6. These examples illustrate the complete process from robot-view input images to detected license plate crops and final recognized plate strings, providing a visual complement to the quantitative results in Table 6.

To further examine the external generalization ability of the detector, an external validation experiment was conducted on a non-overlapping subset selected from CCPD. The subset contains 200 license plate images and was used only for evaluation. Exact file-hash checking found no duplicated images between the external CCPD subset and the EDRV-LP dataset. On this external subset, YOLOv8n achieved a precision of 0.9996, a recall of 1.0000, a mAP_50_ of 0.9950, and a mAP_50:95_ of 0.7997. Under the same setting, YOLOv8-MGL achieved a precision of 0.9997, a recall of 0.9950, a mAP_50_ of 0.9950, and a mAP_50:95_ of 0.8017. These results show that YOLOv8-MGL maintains high detection performance beyond the self-built EDRV-LP dataset.

The supplementary detection–recognition pipeline was also evaluated on the external CCPD subset. As shown in Table 6, YOLOv8-MGL successfully detected 199 out of 200 images. Using the detected crops, the YOLOv8-MGL + CRNN-CTC pipeline achieved an exact plate recognition accuracy of 0.920 over all 200 images and a character-level accuracy of 0.987. These external end-to-end results provide additional evidence for the generalization ability of the detector and the supplementary pipeline.

The remaining recognition errors mainly come from province abbreviation confusion, visually similar alphanumeric characters, and local ambiguity caused by blur or low contrast. These failure cases indicate that, after reliable plate localization is achieved, the main bottleneck of the complete pipeline lies in fine-grained character discrimination under degraded visual conditions.

Overall, the added end-to-end and external validation experiments provide more direct evidence for practical system-level behavior than independent module-level evaluation alone. Nevertheless, the external subset is still limited in scale, and the CPU-only evaluation was conducted on a server-class CPU rather than a dedicated embedded robotic processor. Therefore, future work will further validate the framework on practical embedded robotic hardware, expand evaluation to larger multi-scene datasets, and investigate tighter optimization between YOLOv8-MGL and SimAM-LPRNet in a unified end-to-end detection–recognition pipeline.

## 5. Conclusions

This paper proposed a lightweight robot-view visual sensing framework for license plate detection and recognition in CPU-oriented mobile robotic scenarios. The framework is built around a YOLOv8-MGL detector and a SimAM-enhanced LPRNet recognizer, aiming to address scale-varying license plate localization, dynamic visual degradation, model complexity, and CPU-only inference feasibility.

For license plate detection, YOLOv8-MGL was developed by introducing GhostC2f into the feature aggregation neck of YOLOv8n and inserting LSKAlite after the SPPF module. On the held-out EDRV-LP test set, YOLOv8-MGL achieved 0.995 mAP_50_ and 0.711 mAP_50:95_. Compared with YOLOv8n, it reduced the number of parameters from 3.01 M to 2.77 M and decreased the computational cost from 8.1 to 7.5 GFLOPs, while improving mAP_50:95_ from 0.687 to 0.711. These results indicate that the proposed detector improves the trade-off between model compactness and stricter localization quality compared with YOLOv8n. In comparison with other lightweight and larger-scale detectors, YOLOv8-MGL should be interpreted as a CPU-oriented trade-off solution rather than a detector that maximizes every individual accuracy metric.

For license plate recognition, SimAM was embedded into LPRNet to enhance discriminative character responses without introducing additional learnable parameters. The recognition results on cropped plate images show that SimAM-LPRNet improves the module-level recognition accuracy from 83.10% to 87.17%, demonstrating the effectiveness of parameter-free attention for lightweight recognition under degraded robot-view imaging conditions.

Additional system-level validation with detected crops and an additional CTC-based sequence recognizer was also conducted. On the held-out EDRV-LP test set, this supplementary end-to-end pipeline achieved an exact plate recognition accuracy of 0.910 and a character-level accuracy of 0.981. On the non-overlapping external CCPD subset, it achieved an exact plate recognition accuracy of 0.920 and a character-level accuracy of 0.987. In CPU-only inference, the YOLOv8-MGL detector achieved 23.98 FPS, while the supplementary complete detection–recognition pipeline achieved 13.25 FPS on an Intel Xeon Platinum 8260C platform. These results provide additional system-level evidence for the CPU-oriented feasibility of the proposed framework.

Nevertheless, the current CPU evaluation was conducted on a server-class CPU rather than a dedicated embedded robotic processor, and the dataset scale remains limited. Future work will further validate the framework on practical embedded robotic hardware, expand the dataset to more complex weather and illumination conditions, and investigate tighter optimization between YOLOv8-MGL and SimAM-LPRNet in a unified end-to-end detection–recognition pipeline.

## Figures and Tables

**Table 1:** Detection performance comparison on the held-out EDRV-LP test set.

Model	Params/M	GFLOPs	P	R	mAP_50_	mAP_50:95_
YOLOv5n-u	2.51	7.18	1.000	1.000	0.995	0.712
YOLOv8n	3.01	8.10	0.995	0.995	0.995	0.687
YOLOv10n	2.71	8.39	0.998	0.975	0.994	0.712
YOLOv8s	11.14	28.65	0.990	0.995	0.995	0.719
YOLOv8-MGL	2.77	7.50	1.000	1.000	0.995	0.711

**Table 2:** Ablation study of the proposed YOLOv8-MGL detector on the held-out EDRV-LP test set.

Model Variant	Params/M	GFLOPs	P	R	mAP_50_	mAP_50:95_
YOLOv8n baseline	3.01	8.1	0.995	0.995	0.995	0.687
+ LSKAlite only	3.09	8.2	0.999	1.000	0.995	0.710
+ GhostC2f only	2.68	7.4	1.000	1.000	0.995	0.701
YOLOv8-MGL (GhostC2f + LSKAlite)	2.77	7.5	1.000	1.000	0.995	0.711

**Table 3:** Ablation results of different SimAM insertion positions in LPRNet.

Model Variant	Accuracy (%)	Final Loss
Baseline LPRNet	83.10	0.0096
SimAM after the last MaxPool layer	84.90	0.0080
SimAM after the last two MaxPool layers	87.17	0.0066
SimAM after each MaxPool layer	85.64	0.0058
SimAM before the penultimate CBR block	82.63	0.0052

**Table 4:** Comparison between SimAM and SE attention mechanisms in LPRNet.

Model	Accuracy (%)	Final Loss
LPRNet + SimAM	87.17	0.0066
LPRNet + SE	84.95	0.0082

**Table 5:** CPU-only detection efficiency comparison on the held-out EDRV-LP test set.

Model	Params/M	GFLOPs	mAP_50_	mAP_50:95_	Lat./ms	FPS
YOLOv5n-u	2.51	7.18	0.995	0.712	45.30	22.08
YOLOv8n	3.01	8.10	0.995	0.687	44.55	22.45
YOLOv10n	2.71	8.39	0.994	0.712	50.30	19.88
YOLOv8s	11.14	28.65	0.995	0.719	78.50	12.74
YOLOv8-MGL	2.77	7.50	0.995	0.711	41.70	23.98

**Table 6:** End-to-end license plate recognition performance on the held-out EDRV-LP test set and external CCPD subset.

Dataset	Images	Detected	Exact Acc.	Char. Acc.
EDRV-LP test set	200	200/200	0.910	0.981
External CCPD subset	200	199/200	0.920	0.987

## Data Availability

The datasets used and analyzed during the current study are available from the corresponding author upon reasonable request.
